# A ‘second hit’ mouse model for group 2 and 3 pulmonary hypertension: combination of aortic banding and hypoxia exposure

**DOI:** 10.1093/cvr/cvaf147

**Published:** 2025-08-26

**Authors:** Laura K Pallos, Michaela Matthey, Michael Hesse, Bernd K Fleischmann, Wilhelm Röll, Daniela Wenzel

**Affiliations:** Life and Brain Centre, Medical Faculty, Institute of Physiology I, University of Bonn, Venusberg Campus 1, Gebäude 76, Bonn 53127, Germany; Department of Systems Physiology, Medical Faculty, Institute of Physiology, Ruhr University of Bochum, Universitätsstr. 150, Bochum 44801, Germany; Life and Brain Centre, Medical Faculty, Institute of Physiology I, University of Bonn, Venusberg Campus 1, Gebäude 76, Bonn 53127, Germany; Life and Brain Centre, Medical Faculty, Institute of Physiology I, University of Bonn, Venusberg Campus 1, Gebäude 76, Bonn 53127, Germany; Department of Cardiac Surgery, Medical Faculty, University of Bonn, Bonn, Germany; Life and Brain Centre, Medical Faculty, Institute of Physiology I, University of Bonn, Venusberg Campus 1, Gebäude 76, Bonn 53127, Germany; Department of Systems Physiology, Medical Faculty, Institute of Physiology, Ruhr University of Bochum, Universitätsstr. 150, Bochum 44801, Germany

**Keywords:** Pulmonary hypertension, Second hit, RNA-seq

Group 2 pulmonary hypertension (PH) (due to left heart disease) and Group 3 PH (due to lung disease) are the most common and lethal forms of PH.^1,2^ Because not all patients with heart or lung disease develop PH, a ‘second hit’ model was proposed.^3^ This reflects the clinical situation, as many patients are diagnosed with an overlap of Group 2 and 3 PH, suggesting that either heart or lung disease may represent a ‘second hit’ that triggers the disease. We have therefore established a mouse model for combined Group 2 and 3 PH to test the ‘second hit’ hypothesis for these triggers of PH.

First, we established a model for Group 2 PH applying mild transverse aortic constriction (TAC) using a 26-gauge needle in C57BL/6 mice. Analysis was performed after 3 and 8 weeks. Macroscopic images of hearts and heart weight to body weight or tibial length measurements demonstrated increased organ size in the TAC group compared to sham-operated animals. As expected, functional analysis with echocardiography and Millar catheter revealed impaired LV function, an increase in LV systolic pressure (LVSP) (*Figure 1A*), and enhanced cross-sectional cardiomyocyte area (CSA) in the LV at both timepoints. RVSP and CSA in the RV were unaltered 3 weeks after TAC, but after 8 weeks, both parameters increased (*Figure 1B* and *C*), reflecting the time-dependent development of Group 2 PH. Sirius red staining showed fibrotic remodelling in the RV already as early as 3 weeks, which continued to progress through 8 weeks. We also found elevated pulmonary vascular wall thickness and lung fibrosis at both timepoints demonstrating lung remodelling.

**Figure 1:**
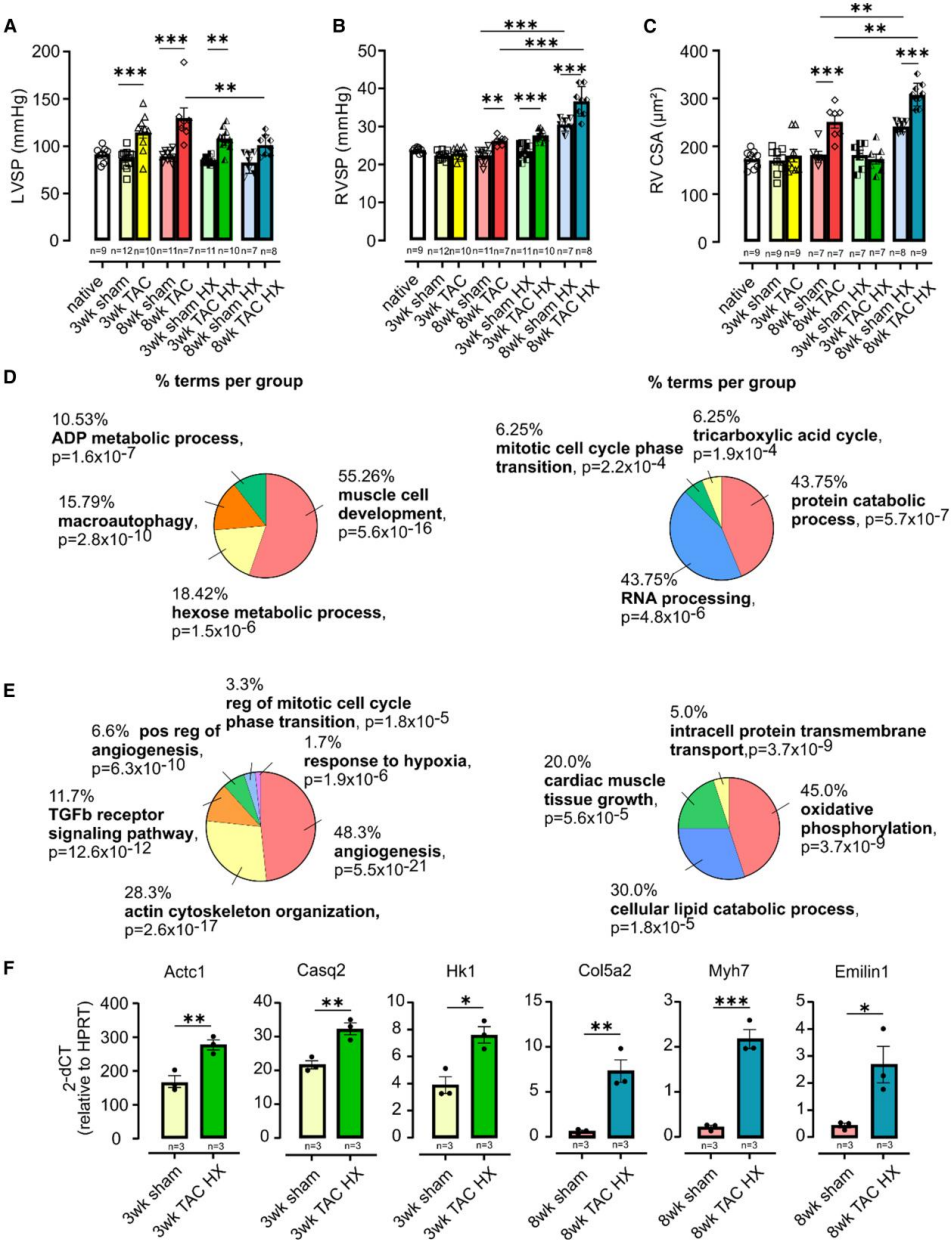
Characterization of a ‘second hit’ mouse model for Group 2 and 3 PH. (*A–C*) Analysis of left ventricular systolic pressure (LVSP, *A*), right ventricular systolic pressure (RVSP, *B*), and RV cardiomyocyte cross-sectional area (CSA, *C*) 3 and 8 weeks after TAC and TAC HX. (*D*, *E*) GO analysis of the uniquely upregulated (left) and downregulated (right) genes in the RV after 3 weeks (*D*) and 8 weeks. (*E*) TAC HX vs. sham (all *n* = 3). (*F*) qPCR analysis of key uniquely upregulated genes in TAC HX. (*A–C*) One-way ANOVA, Tukey’s *post hoc* test. (*F*) Unpaired Student’s *t*-test, *P < 0.05, **P < 0.01, ***P < 0.001.

To develop a ‘second hit’ mouse model for combined Group 2 and Group 3 PH, we exposed animals to mild TAC and additionally to hypoxia (HX, 10% O_2_) during the last 10 days of the 3-week and 8-week protocols. Successful TAC was again reflected by elevated heart weight and compromised LV function in TAC HX vs. sham HX animals.

At 3 weeks, the combination of TAC and HX resulted in elevated RVSP, indicating the development of PH already after 3 weeks, thereby aggravating the effects of TAC alone (*Figure 1B*). LVSP, LV CSA, RV CSA (*Figure 1C*), RV collagen deposition, and lung remodelling were similar to TAC alone.

At 8 weeks, HX during the last 10 days further increased RVSP but reduced LVSP compared to TAC alone (*Figure 1A* and *B*). This suggests an amplified contractile response to elevated afterload in the RV and potential early LV failure, as supported by echocardiography. RV hypertrophy was more pronounced (*Figure 1C*), whereas LV CSA remained unchanged compared to TAC alone. The stronger response in the 8-week sham HX animals compared to the 3-week animals may be related to their older age at the time of the experiment. Interestingly, RV and lung collagen deposition were reduced in TAC HX at 8 weeks compared to TAC alone.

To examine changes in the gene expression pattern of the RV, we performed RNA-seq analysis on sham, TAC, sham HX, and TAC HX animals at 3 and 8 weeks. To characterize the consequences of combined Group 2 and 3 PH on the RV, we analysed the unique genetic alterations of TAC HX vs. sham that were neither regulated in TAC vs. sham nor sham HX vs. sham. At 3 weeks, we found 658 differentially expressed genes (DEGs) uniquely regulated in TAC HX vs. sham (287 upregulated, 371 downregulated), and at 8 weeks, there were 468 DEGs (206 upregulated, 262 downregulated). Gene ontology (GO) analysis revealed upregulation of genes related to muscle development, angiogenesis, hexose metabolism/glycolysis, and response to HX (*Figure 1D* and *E*, left). Genes associated with catabolism, mitosis, and tricarboxylic acid (TCA) cycle/oxidative phosphorylation and also certain genes related to cardiac growth were downregulated (*Figure 1D* and *E*, right). The results were confirmed by qPCR analysis of key genes that are uniquely upregulated by TAC HX after 3 or 8 weeks (*Figure 1F*). These changes illustrate the biological and metabolic processes reflecting cardiac remodelling.

Herein, we propose a ‘second hit’ mouse model for PH by combining mild TAC with HX. Neither of the treatments alone could induce PH within 3 weeks, but their combination resulted in elevated RVSP, indicating the onset of PH. After TAC alone, our findings suggest that the pathomorphological changes occur later in the RV than in the LV, as proposed earlier by Platt *et al*.^4^ in a 26-gauge TAC model, where a delayed increase in RVSP and RV CSA was reported compared to LVSP and LV CSA. Fibrosis in both ventricles in our study correlates with pressure increase and hypertrophy in the LV but precedes the onset of haemodynamic changes in the RV. Such biventricular remodelling has previously been reported after inducing RV pressure overload and was attributed either to the mechanical interdependence of both heart chambers or to hormones and growth factors that affect the whole heart.^5,6^ There was no synergistic effect of TAC and HX on RV and lung collagen deposition, but a reduction after 8 weeks, possibly due to anti-remodelling effects of HX, as reported at least for the LV.^7^ Nevertheless, the RV genetic profile after TAC HX reflects a stress response, with upregulation of HX/HIF-1a-related genes, predicting pathological remodelling at 3 weeks and indicating established remodelling or hypertrophy at 8 weeks.^8,9,10^ Thus, we demonstrate the establishment of a mouse model for Group 2 and 3 PH, where heart or lung disease acts as a ‘second hit’ for PH development.

All animal experiments were conducted in compliance with the National Institutes of Health animal protection guidelines and approved by the local authorities [Landesamt für Natur, Umwelt und Verbraucherschutz, Nordrhein-Westfalen (LANUV), NRW, Germany].

For TAC surgery 30 min before the intervention, mice were injected with buprenorphine (0.1 mg/kg s.c.) for analgesia. Then, animals were anaesthetized by inhalation of 4–5% isoflurane and intubated with a 22 G needle. For maintenance of anaesthesia, isoflurane levels were reduced to 1.0–2.5%. For postoperative analgesia, buprenorphin (0.1 mg/kg s.c.) was injected twice a day and applied via the drinking water (1 mg/kg) overnight for 3 days. For transaortic echocardiographic analysis, mice were anaesthetized with 4–5% isoflurane, and for maintenance, 1.0–1.5% isoflurane was applied. For haemodynamic analysis, mice were anaesthetized with 4–5% isoflurane, intubated, ventilated, and placed on a heating plate (37°C). Then, ketamine (50 mg/kg) and xylazine (5 mg/kg) were applied i.p. For maintenance of anaesthesia, isoflurane levels were reduced to 1–1.5%. After the haemodynamic measurements, mice were sacrificed by cervical dislocation.

## Authors’ contributions

L.K.P. performed animal and catheter measurements as well as echocardiography and histology and analysed data and contributed to the writing of the manuscript. M.M. analysed histological sections. M.H. performed RNA-seq analysis. B.K.F. discussed data and contributed to the writing of the manuscript. W.R. supervised animal experiments and analysis. D.W. designed the study, supervised the experiments, and wrote the manuscript.

## Data Availability

All data associated with this study are present in the paper or available from the corresponding author upon request. All sequencing data sets reported in this manuscript are deposited in the Short Read Archive at the National Center for Biotechnology Information under the BioProject ID XX.
